# Genome-Based Retrospective Analysis of a *Providencia stuartii* Outbreak in Rome, Italy: Broad Spectrum IncC Plasmids Spread the NDM Carbapenemase within the Hospital

**DOI:** 10.3390/antibiotics12050943

**Published:** 2023-05-22

**Authors:** Valerio Capitani, Gabriele Arcari, Alessandra Oliva, Federica Sacco, Gaia Menichincheri, Linda Fenske, Riccardo Polani, Giammarco Raponi, Guido Antonelli, Alessandra Carattoli

**Affiliations:** 1 Department of Public Health and Infectious Diseases, Sapienza University of Rome, 00185 Rome, Italy; 2 Department of Molecular Medicine, Sapienza University of Rome, 00161 Rome, Italy; 3 Bioinformatics and Systems Biology, Justus-Liebig-University Giessen, 35390 Giessen, Germany

**Keywords:** *Enterobacterales*, antibiotic resistance, IncC plasmid, opportunistic pathogen, *Providencia stuartii*, plasmid mediated resistance, *bla*_NDM_

## Abstract

*Providencia stuartii* is a member of the *Morganellaceae* family, notorious for its intrinsic resistance to several antibiotics, including last-resort drugs such as colistin and tigecycline. Between February and March 2022, a four-patient outbreak sustained by *P. stuartii* occurred in a hospital in Rome. Phenotypic analyses defined these strains as eXtensively Drug-Resistant (XDR). Whole-genome sequencing was performed on the representative *P. stuartii* strains and resulted in fully closed genomes and plasmids. The genomes were highly related phylogenetically and encoded various virulence factors, including fimbrial clusters. The XDR phenotype was primarily driven by the presence of the *bla*_NDM-1_ metallo-β-lactamase alongside the *rmtC* 16S rRNA methyltransferase, conferring resistance to most β-lactams and every aminoglycoside, respectively. These genes were found on an IncC plasmid that was highly related to an NDM-IncC plasmid retrieved from a ST15 *Klebsiella pneumoniae* strain circulating in the same hospital two years earlier. Given its ability to acquire resistance plasmids and its intrinsic resistance mechanisms, *P. stuartii* is a formidable pathogen. The emergence of XDR *P. stuartii* strains poses a significant public health threat. It is essential to monitor the spread of these strains and develop new strategies for their control and treatment.

## 1. Introduction

The *Providencia* species has historically been described as a member of the *Proteeae* tribe [1]. Following a classification proposal for genera previously assigned to the *Enterobacteriaceae* family, the genus *Providencia* is now considered to belong to the *Morganellaceae* family in the *Enterobacterales* order [2]. From a biochemical viewpoint, bacteria belonging to this family are negative for oxidase, arginine decarboxylase, and Voges–Proskauer.

*Providencia stuartii* is an opportunistic pathogen capable of causing infection mainly of the respiratory and urinary tract [3], and it contributes significantly to biofilm formation in catheterized patients, also causing relevant outbreaks in hospital settings [3,4,5,6].

In the current antimicrobial resistance (AMR) crisis [7], bacteria belonging to the *Morganellaceae* family should be regarded as dangerous foes, displaying an intrinsic resistance to antibiotics such as colistin (due to the carriage of the *arnBCADTEF* and *eptB* genes, whose expression results in the addition of phosphoethanolamine and arabinose cationic groups on the Lipopolysaccharide, respectively [8]) and tigecycline [9], coupled with higher MICs for the carbapenem imipenem [10]. The use of the colistin and tigecycline as last-resort antimicrobial agents to treat Multi-Drug-Resistant (MDR) [11] strains is significantly promoting the spread of members of the *Morganellaceae* family [12].

Numerous cases of *P. stuartii* carrying relevant antibiotic resistance genes, located either on the chromosome or on plasmids, have been described. *P. stuartii* isolates carrying chromosomal-located *bla*_NDM-1_ and *bla*_OXA-48_ carbapenemase genes were identified in Switzerland in wound samples from a patient transferred from Macedonia, and several isolates carrying carbapenemases, such as KPC and VIM, have been described in Greece, Argentina, Brazil, and Saudi Arabia [6,13,14,15,16]. In recent years, a large number of carbapenem-resistant *P. stuartii* carrying *bla*_NDM_ has been reported [3,15,17,18]. Several sequences of IncC-type plasmids carrying *bla*_NDM_ isolated in *P. stuartii* strains are available [12,13]. These data raise attention to the relevant role of this microorganism in the spread of antimicrobial resistance genes.

This study reports the characterization of *P. stuartii* causing an outbreak in two Intensive Care Units (ICUs) at the Policlinico Umberto I Hospital in Rome (PUI). This outbreak was noteworthy because *P. stuartii* is a relatively uncommon pathogen and *bla*_NDM_, with the exception of an outbreak in Tuscany [19,20], is rarely reported in Italy [21]. Given the eXtensively Drug-Resistant (XDR) phenotype these isolates displayed, being susceptible only to the trimethoprim-sulfamethoxazole combination and aztreonam, a genomic investigation was conducted. The objectives of these analyses were: (1) to describe the outbreak strain; (2) to identify the genetic mechanisms at the basis of the *bla*_NDM_ acquisition; (3) to detect if other relevant resistance mechanisms were co-acquired alongside the *bla*_NDM_; and (4) to compare Italian isolates at a global level. The study outcome was to gain a deeper knowledge about the diffusion and spread of an emerging MDR-resistant pathogen, *P. stuartii*.

## 2. Results and Discussion

### 2.1. An Outbreak of NDM-Producing Providencia Stuartii

Between February and March of 2022, four cases of colonization or infection sustained by NDM-producing *P. stuartii* were identified at PUI, and a total of seven isolates were collected from four patients (Table 1). 

All patients were males, with a median age of 51 years and a median Intensive Care Unit (ICU) stay of 35 days. Two of them were hospitalized within the 90 days prior to the *P. stuartii* diagnosis. Within the 90 days before hospitalization, all patients received antibiotic therapy, but none of the antibiotics used were based on a carbapenem regimen (Table S1).

Antimicrobial susceptibility testing (AST) revealed that the strains were resistant to all tested β-lactams, except monobactams (ceftazidime, piperacillin/tazobactam, cefoxitin, cefuroxime, ceftazidime/avibactam, ceftolozane/tazobactam, imipenem, meropenem), aminoglycosides (amikacin), ciprofloxacin, and fosfomycin, but susceptible to co-trimoxazole (Table 1). In addition, *P. stuartii* is intrinsically resistant to colistin, tigecycline, gentamicin, and tobramycin.

Patients #3 and #4, who had a respiratory tract infection sustained by NDM-producing *P. stuartii*, were treated with the combination of aztreonam (AZT) + ceftazidime/avibactam (CZA) (Table S1). The rationale of this therapeutic regimen is that AZT is stable against Metallo-β-lactamases such as NDM, yet these strains may also encode for Extended Spectrum β-lactamases (ESBLs) and/or AmpC enzymes, which may hydrolyze AZT; thus, AZT alone has limited clinical utility against NDM-producing strains. The avibactam component of CZA, instead, has no ability to inhibit metallo-β-lactamases but can protect AZT from the hydrolysis of secondary ESBLs or AmpCs [22].

Of the seven isolates, three prototypical isolates (namely 65, 883, and 41) sampled from three different sources (urinary tract, respiratory tract, and rectal swab) in three different patients from two wards, were chosen to represent the complexity of this outbreak. The three strains underwent both Illumina and Nanopore sequencing.

All genomes were positive for the *aac(6′)-Ib3, sul1, ΔqacE, rmtC*, *bla*_NDM-1_, and *bla*_CMY-6_ genes located on IncC plasmids and the *tet(B), catA3* and *aac(2′)-Ia* genes identified in the chromosome. Furthermore, isolate 41 carried a ColpVC plasmid encoding no resistance.

### 2.2. The NDM-Carrying IncC Plasmid

The first plasmid attributed to the IncC group (R16a) can be traced back to 1966 and was identified in a *P. stuartii* strain [23], suggesting a long-term relationship between IncC and the *Providencia* genus. However, in more recent years, some MDR plasmids have been reported in *Providencia* strains; among them, there are several IncC-type plasmids carrying *bla*_NDM_ isolated from *P. stuartii* strains that have been described [17,18].

In the three sequenced *Providencia* isolates from our hospital, a 141,594-bp IncC plasmid was identified, highly conserved among them (100% identity, 100% coverage).

We compared one of these plasmids, belonging to isolate 41 (pVCpro_41, Acc. No. OQ750828), with another *bla*_NDM_-carrying IncC plasmid from *Klebsiella pneumoniae* isolate 0831 (p0831_NDM_IncC, Acc. No. MZ606383), sampled from our hospital in 2020 [24].

**Figure 1:**
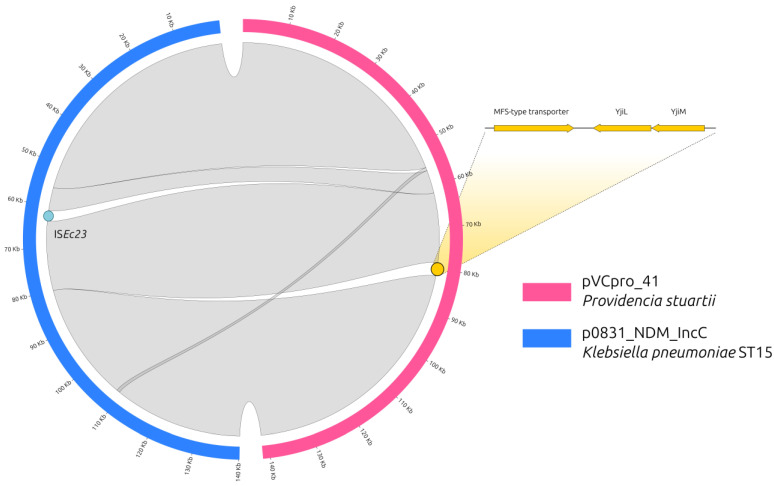
Circos plot of two *bla*_NDM_-carrying IncC plasmids. Circos plot representing the synteny between two *bla*_NDM_-carrying IncC plasmids, pVCpro_41, and p0831_NDM_IncC, isolated from *Providencia stuartii* and a *Klebsiella pneumoniae,* respectively. The isolates were sampled from the Policlinico Umberto I University Hospital of Rome, Italy. Half circles represent the plasmids, color-coded according to the legend. The gray bands depict homology regions with 100% sequence identity between the plasmids, while white areas represent discontinuities (i.e., an IS*Ec*23 in the case of the blue dot and the three genes represented by yellow arrows in the case of the yellow dot).

These two plasmids were almost identical, having a percentage of identity of 100% (Figure 1), but differed in length. 

It was possible to observe the following:

- p0831_NDM_IncC carried an Insertion Sequence (IS, IS*Ec*23), which was not present in the *P. stuartii* plasmid;
- pVCpro_41 carried three genes, which were not present in the p0831_NDM_IncC plasmid, encoding for an uncharacterized MFS-type transporter, and two uncharacterized proteins named YjiL and YjiM.

Performing a BLASTN search of the MFS-type transporter, *yjiL* and *yjiM* genes in the NCBI database, all results were from the chromosome of isolates belonging to the *Klebsiella* genus; on excluding this genus from the search, no perfect match (100% coverage, 100% identity) was obtained. No other IncC plasmid in the PLSDB, including those identified in *Klebsiella*, carried these extra genes in its scaffold. These three genes were also found in the chromosomes of NDM-producing ST15 *K. pneumoniae* strains involved in an outbreak that occurred in 2020 in the same hospital (BioProject PRJNA746265) [24]. Furthermore, NDM-IncC plasmids were not detected in any other bacterial strain isolated at the hospital in the period of 2020–2022.

Despite *P. stuartii* isolates expressing both NDM, CMY-2 and RmtC, have already been described in Egypt [10]; the presence of the three genes from the *Klebsiella* chromosome strongly supports the hypothesis that the IncC plasmid, before being transferred into *P. stuartii*, passed through ST15 outbreak *K. pneumoniae* strains. Nonetheless, before the *P. stuartii* outbreak in 2022, it is possible to speculate that this IncC plasmid was maintained in the hospital in hidden reservoirs for almost two years, probably from environmental sources.

### 2.3. Genomic Epidemiology of bla_NDM_-Carrying IncC Plasmids

IncC plasmids are commonly found in multi-drug-resistant Gram-negative bacteria of various species, indicating the wide host range of these plasmids. A total of 534 plasmids containing the IncC replicon, found in 18 different bacterial genera, were obtained from the PLSDB database [25] and screened using Kleborate [26]. Of these, 89 carried the *bla*_NDM_ gene, mainly in its NDM-1 allele (84/89).

Due to the high levels of correlation between *bla*_NDM_ gene presence and 16S rRNA methyltransferase (16RMTases) gene co-presence on the same plasmid, IncC plasmids carrying *bla*_NDM_ have a higher likelihood of carrying a 16RMTases gene (39.8% vs. 10.1%, *p* < 0.0001). Instead, there are no significant differences between IncC plasmids carrying *bla*_NDM_ and the ones not carrying it, both in terms of length (mean 162.1 kb vs. 162.9 kb) and coding sequences (CDSs, mean 206.4 of which 62 are “core” vs. 199.4 of which 30 are “core”), defining a specific IncC plasmid Core Genome (pCG). 

**Figure 2:**
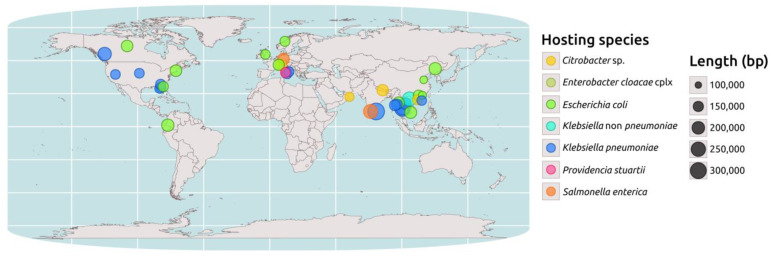
Geospatial analysis of the *bla*_NDM_-carrying IncC plasmids. Map representing the distribution of a selected set of 39 IncC plasmids carrying the *bla*_NDM_ gene retrieved from the PLSDB database, for which geographical coordinates were available. Additionally, the three *Providencia stuartii* (pink dot) plasmids described in this study and the two found in ST15 *Klebsiella pneumoniae* (blue dot) in the same hospital were added to the map. Plasmid sequences were screened through the Kleborate tool. The map was created using the “maps” and “ggplot2” packages in the R programming language. The circles in the map represent the IncC plasmids, which are color-coded based on the hosting species. The size of the circles is proportional to the plasmid size.

The PLSDB database [12] shows that complete IncC plasmids encoding for NDM are exclusively found within the *Enterobacteriaceae* family, with *Escherichia coli* and *K. pneumoniae* being the most identified host species (31 and 27 isolates, respectively). A geospatial analysis performed on a subset of 44 NDM-encoding IncC plasmids, 39 from PLSDB with available coordinates, 3 from *P. stuartii,* and 2 from *K. pneumoniae* identified at the PUI, revealed that their distribution is not uniform. These plasmids are more widely distributed in Southeast Asia, Central Europe, and North America, with a greater prevalence in these regions (Figure 2).

To investigate the relationship between the pCG in the NDM-encoding IncC plasmids and the species carrying them, a phylogenetic analysis was conducted on the above-mentioned 89 plasmids, as well as on the 3 additional IncC plasmids obtained from *P. stuartii* in the current study and the 2 IncC plasmids from the ST15 *K. pneumoniae* previous study, performed in the same hospital [24]. 

**Figure 3:**
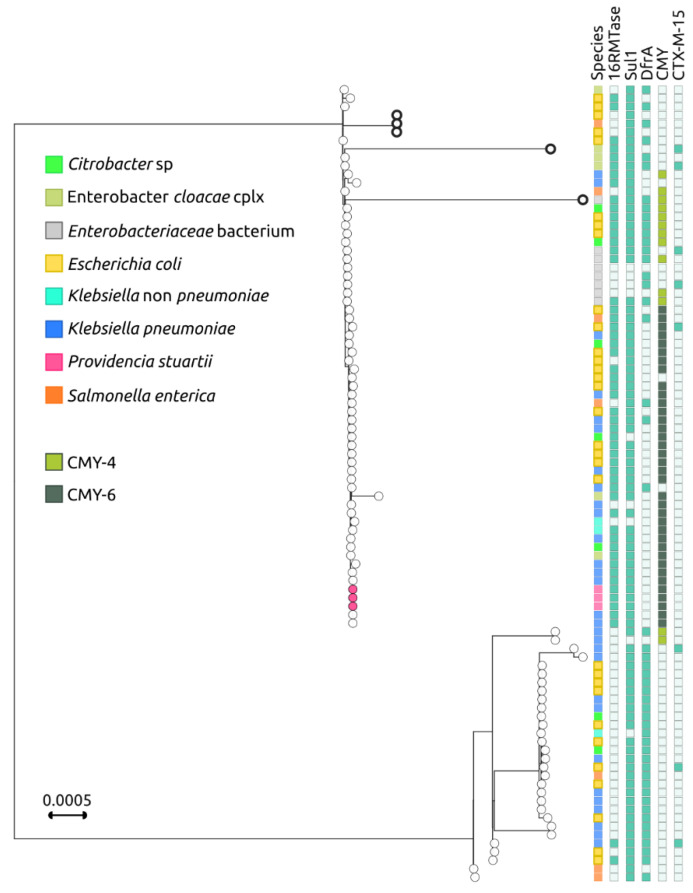
Phylogenetic tree of NDM-encoding IncC plasmids based on plasmid Core genome (pCG). Phylogenetic tree of 93 IncC plasmids (89 retrieved from the PLSDB database, 3 from this study, and plasmids p0831_NDM_IncC (MZ606383) and p1027_NDM_IncC (MZ606384) from ST15 *K. pneumoniae* isolated in our hospital carrying the *bla*_NDM_ carbapenemase, based on the concatenation of the 60 core genes, which constitute the plasmid Core genome (pCG). Metadata colors depicting the hosting species (first column) and the CMY variant (fifth column) are explained in the legend. The three IncC plasmids identified in the isolates sequenced in this study are indicated by magenta dots. The five divergent IncC plasmids in the most populated branch, discussed in the text are represented by bold circles.

This analysis divided the NDM-encoding IncC plasmids into two main branches. The first branch included the *P. stuartii* plasmids from this study, which clustered together with 59 closely related plasmids (Figure 3). 

The first branch, except for a small cluster of three plasmids from Southeast Asia (CP031297 found in *E. coli* from Vietnam, MN604267 found in *Salmonella enterica* serovar London, and MN604268 found in *E. coli* from Singapore [27]) and two highly divergent plasmids (CP041052 found in *Enterobacter hormaechei* and MN657252 found in an *Enterobacteriaceae* [28]), showed an extremely well-conserved pCG. The second branch is less populated and composed of 30 plasmids, which were found to be more heterogeneous in terms of pCG. Additionally, 28 out of the 30 plasmids in this branch did not have genes encoding for 16RMTases, and 28 out of the 30 did not encode for CMY. IncC plasmids are one of the main routes for the diffusion of *bla*_CMY_ cephalosporinase genes [29], which, in association with *bla*_NDM_, confer resistance to most β-lactams. Overall, the findings suggest that the NDM-encoding IncC plasmids are diverse in terms of their pCG and host species. The first branch of plasmids appears to be highly conserved, while the second branch is less conserved and lacks the 16RMTases gene in most cases; both plasmid lineages can be hosted by multiple species (Figure 3).

### 2.4. Anatomy of Three Providencia Stuartii Isolates Carrying the IncC Plasmid

Given the high conservation of the IncC plasmids among them and with those identified in ST15 *K. pneumoniae*, an in-depth characterization of the three *P. stuartii* strains was needed to discern between horizontal transfer of IncC in three different *P. stuartii* recipients and vertical expansion of one single *P. stuartii* NDM-positive clone.

The three sequenced isolates from PUI have highly similar genomes, differing by 2–5 SNPs on 4120 core genes (Figure S1, dataset File S1). One of these SNPs differentiated the isolate 41 from isolates 65 and 883, causing a frameshift variant of the efflux transporter periplasmic adaptor sub-unit MexH, often implied in AMR in members of the *Pseudomonas* genus [30]. The contribution of this mutation to the AMR profile could not be evaluated due to the substantial number of resistance genes already carried by these isolates.

A total of 17 different virulence-related genes were identified, encoding for the biosynthesis of the O-antigen and LPS (*pgi, lpxC, msbA, lpxB, galE, rmlB, wecA*), the flagellum (*fliG, flgG, flgE, cheA*), and the numerous secretion systems present in the *P*. *stuartii* genome (*ysaC* and *escR* genes, belonging to a type 3 secretion system, and four copies of the *hcp-2* gene for the type 6 secretion system). Several gene clusters belonging to the chaperone-usher fimbriae and crucial virulence factors [31] were identified in the sequenced strains. 

**Figure 4:**
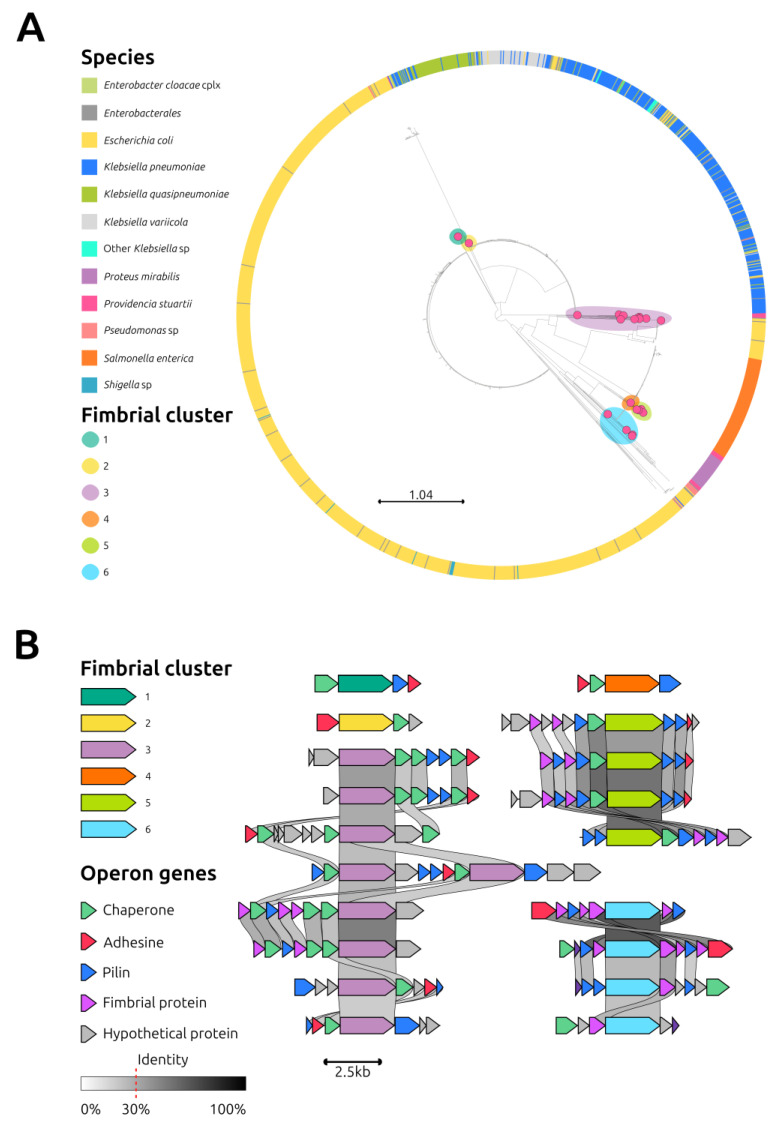
Analysis of fimbrial clusters. (**A**): Phylogenetic analysis of 1881 usher proteins (1860 downloaded from the RefSeq NCBI Protein DataBase, https://www.ncbi.nlm.nih.gov/protein, accessed on 31 January 2023, using as query “Fimbria” AND “Usher” and 21 identified in isolate 41). Metadata colors depicting the hosting species are explained in the legend. The 21 usher proteins identified in isolate 41 are indicated by magenta dots; the colored halos around magenta dots indicate the corresponding fimbrial cluster. (**B**): Synteny analysis of the operons belonging to the 6 fimbrial clusters identified in *Providencia stuartii* isolate 41. Arrows represent genes and are color-coded according to the legend. Grey links represent the nucleotide homology between genes. The nucleotide identity threshold was set to 30%, and higher identity is reflected by a darker shade of gray.

Overall, 21 ushers were identified in the 3 sequenced strains of *P. stuartii*. These protein sequences were compared to those from other species available in the GenBank database. The resulting phylogenetic tree showed that the ushers from *P. stuartii* clustered into six distinct branches (Figure 4A). Except for one usher in fimbrial cluster 4, which was also found in *Proteus mirabilis*, all other ushers were specific to *P. stuartii*. Conducting a detailed analysis of these clusters, significant variations even within the same phylogroup were detected (Figure 4B).

A CAS-TypeIF bacterial defense system, located within the cluster encoding for the flagellum, was identified. Nonetheless, not all the CRISPR regions were located within the flagellar cluster, with some dispersed in other sites of the genome. Furthermore, only two of the identified spacers could be associated with bacteriophages.

Three prophages could be localized in the analyzed *P. stuartii* chromosomal sequences; specifically, one belonged to the *Myoviridae* family, one to the *Siphoviridae* family, and one could not be clearly classified.

## 3. Materials and Methods

### 3.1. Bacterial Isolation and Antimicrobial Susceptibility Testing

Between February and March 2022, seven *Providencia stuartii* isolates were retrieved from four patients at the PUI (Table 1).

Bacteria were isolated from samples collected during routine microbiologic processes. Isolated colonies were identified as *P. stuartii* by matrix-assisted laser desorption ionization-time of flight mass spectrometry (MALDI-TOF MS) system (Bruker Daltonics GmbH, Bremen, Germany). The antimicrobial susceptibility test was carried out using the MicroScan WalkAway system (Beckman Coulter, Inc., Brea, CA, USA), testing the following antibiotics: amikacin, gentamicin, tobramycin, piperacillin/tazobactam cefuroxime, ceftazidime, cefoxitin, ceftazidime/avibactam, ceftolozane/tazobactam, imipenem, meropenem, ciprofloxacin, co-trimoxazole, fosfomycin, colistin, and tigecycline.

### 3.2. Whole-Genome Sequencing and Assembly

Among the seven above-mentioned isolates, three prototypical strains (namely 41, 65, and 883) isolated from three patients (Table 1) were further characterized by Whole-Genome Sequencing (WGS).

Genomic DNA extracted using the Bioline kit was used as input for the Illumina MiSeq instrument (Illumina, Inc., San Diego, CA, USA), following the Nextera XT DNA sample preparation kit, which generated paired-end libraries with the 2 × 300 PE protocol (Illumina). High-molecular-weight genomic DNA was purified using a proteinase K-phenol-chloroform extraction method and used for Oxford Nanopore Technologies (ONT) sequencing, as previously described [32]. Illumina reads and ONT assemblies were integrated by the Unicycler tool version 0.4.8.0 using a bold bridging mode [33].

### 3.3. Genomic and Phylogenetic Analyses

The mobilome and resistome of the three *P. stuartii* genomes sequenced in this study were, respectively, analyzed using PlasmidFinder [34] and ResFinder [35] at the Center for Genomic Epidemiology website (http://www.genomicepidemiology.org/services/ (accessed on 31 May 2022)).

Their genomes were annotated using RAST [36] and Prokka [37]. The presence of CRISPR loci was assessed by CRISPR-Cas finder [38] and the ones of phages by Phigaro [39].

Manual curation was performed on the annotated genomes to identify the genes encoding for fimbrial clusters using the EDGAR 3.0 server [40] and Bakta [41]. To classify and evaluate the identified fimbriae, a comparative analysis of the amino acid sequence of the fimbrial usher proteins of isolate 41 was performed in comparison with those obtained from the NCBI Protein Database. Specifically, a phylogenetic relatedness analysis of the fimbrial usher protein [42] was performed with 1860 usher proteins and the relative metadata downloaded from the RefSeq NCBI Protein Database (https://www.ncbi.nlm.nih.gov/protein (accessed on 31 January 2023)) using as query “Fimbria” AND “Usher”. The synteny among the identified fimbrial operons was visualized using the Clinker tool [43] and adjusted using the open-source InkScape software.

A total of 60 genomes belonging to *P. stuartii* (3 sequenced in this study and 57 downloaded from the NCBI database) were annotated using Prokka [37]. A core-gene alignment was built using Roary [44] from the respective GFF, with a minimum percentage identity for BLASTP of 95% and a percentage of isolates a gene must be in to be core of 95%. A consensus phylogenetic tree based on 1000 ultrafast bootstraps [45] was generated with IQ-TREE [46] using the GTR + F + I + G4 substitution model. All phylogenetic analyses were carried out using the Galaxy Europe instance (https://usegalaxy.eu/ (accessed on 28 February 2023)). The phylogenetic tree and its metadata were visualized using Microreact [47] and adjusted using the open-source InkScape software. To enhance the clarity of the tree, 10 genomes downloaded from the GenBank database were excluded from its final version because their significant phylogenetic distance made it harder to interpret the relationships between our isolates and the rest of the tree.

The analysis of virulence genes was carried out using the Virulence Finder Database [48], taking into account genes with a percent identity and coverage greater than 70%.

The R programming language (Version 4.2.2) and two related packages (maps and ggplot2) were utilized to obtain world map data using the ‘map_data’ function from the maps package.

### 3.4. IncC Plasmids Analysis

Plasmids carrying the IncC replicon were downloaded from the PLSDB [25] database version 2021_06_23_v2, filtered for the presence of the PlasmidFinder [34] IncC replicon and screened for the presence of *bla*_NDM_ using Kleborate [26]. A phylogenetic analysis based on a core-gene alignment of the *bla*_NDM_-positive IncC plasmid was performed using Roary [44] from the respective GFF, with a minimum percentage identity for BLASTP of 90% and a percentage of isolates a gene must be in to be core of 95%. The resulting core genome has been defined as the IncC plasmid Core Genome (pCG). A consensus phylogenetic tree based on 1000 ultrafast bootstraps [45] was generated with IQ-TREE [46] using the GTR + F + G4 substitution model. The tree and metadata were visualized using Microreact [47] and adjusted using the open-source InkScape software verison 1.2.2.

### 3.5. Statistical Analyses

Statistical analyses were performed using JASP version 0.17.1. To assess the relationships between categorical variables and to determine differences between continuous variables, χ^2^ and Mann–Whitney U tests were deployed, respectively. The χ^2^ test compares observed and expected frequencies to determine if there is a significant association between the two categorical variables. The Mann–Whitney U test compares the medians of two independent samples to determine if they come from the same population or not. Both tests were used to evaluate the significance of the results at *p* < 0.01.

## 4. Conclusions

A small outbreak sustained by NDM-1-producing *Klebsiella pneumoniae* occurred in 2020 at the PUI in Rome. In those isolates, we identified an IncC plasmid carrying the *bla*_NDM_ and *rmtC* genes. This plasmid was not reported in other strains from this hospital in the following two years. In 2022, an NDM-1-encoding IncC plasmid carrying three genes from the *K. pneumoniae* chromosome was identified in *Providencia stuartii*, causing a small outbreak in the hospital.

The presence of an IncC plasmid harboring *bla*_NDM_ in a member of the *Morganellaceae* family, intrinsically resistant to several last-resort antibiotics such as colistin or tigecycline, is of the uttermost relevance.

The limitation of this study is the small number of outbreak isolates and the lack of intermediate strains demonstrating the hypothesis of a passage of *bla*_NDM-1_ IncC plasmids between *K. pneumoniae* and *P. stuartii.* However, the IncC plasmid of *P. stuartii* acquired chromosomal genes from *K. pneumoniae*, and these genes were not present in other IncC plasmids, suggesting that horizontal transfer between the two species occurred.

## Figures and Tables

**Table 1:** Antimicrobial susceptibility testing and sampling origin of the *Providencia stuartii* strains isolated in this study. Values in bold indicate resistance.

PT	ID	SS	MIC mg/L												
			AK	P/T	CAZ	FOX	CRM	CZA	C/T	IMI	MEM	CIP	SXT	AZT	FOS
Pt#1	41 *	RS	**32**	**>16**	**>32**	**>16**	**>8**	**>16**	**>32**	**>16**	**>16**	**>4**	80	**≤1**	**>64**
Pt#2	**38**	RS	**32**	**>16**	**>32**	**>16**	**>8**	**>16**	**>32**	**>16**	**>16**	**>4**	40	**2**	**>64**
Pt#3	883 *	TBA	**32**	**>16**	**>32**	**>16**	**>8**	**>16**	**>32**	**>16**	**>16**	**>4**	40	**≤1**	**>64**
	**919**	UR	**32**	**>16**	**>32**	**>16**	**>8**	**>16**	**>32**	**>16**	**>16**	**>4**	40	**≤1**	**>64**
Pt#4	65 *	UR	**32**	**>16**	**>32**	**>16**	**>8**	**>16**	**>32**	**>16**	**>16**	**>4**	40	**≤1**	**>64**
	**607**	TBA	**32**	**>16**	**>32**	**>16**	**>8**	**>16**	**>32**	**>16**	**>16**	**>4**	40	**2**	**>64**
	**317**	TBA	**32**	**>16**	**>32**	**>16**	**>8**	**>16**	**>32**	**>16**	**>16**	**>4**	40	**≤1**	**>64**

PT: Patient; ID: Sample name; SS: Sample Source; RS: Rectal Swab; TBA: Tracheo-Bronchial Aspirate; UR: Urine; AK: Amikacin; P/T: Piperacillin/Tazobactam; CZA: Ceftazidime; FOX: Cefoxitin; CRM: Cefuroxime; CZA: Ceftazidime/avibactam; C/T: Ceftolozane/tazobactam; IMI: Imipenem; MEM: Meropenem; CIP: Ciprofloxacin; SXT: Co-trimoxazole; AZT: Aztreonam; FOS: Fosfomycin; Tigecycline and Colistin were not reported since *P. stuartii* is intrinsically resistant to these antibiotics. * Sample subjected to Illumina and Oxford Nanopore Technologies Sequencing.

## Data Availability

1. A BioProject has been released at DDBJ/ENA/GenBank, no.: PRJNA948429 (https://www.ncbi.nlm.nih.gov/sra/PRJNA948429, accessed on 1 May 2023); 2. Circular complete pVCpro_41 plasmid has been released under accession no. OQ750828.

## Supplementary Materials

The following supporting information can be downloaded at: https://www.mdpi.com/article/10.3390/antibiotics12050943/s1, Figure S1: Phylogenetic tree of *Providencia stuartii*; Table S1: Clinical information. File S1: Supplementary Dataset.
