# Supplementary material for: Genome-Based Retrospective Analysis of a Providencia stuartii Outbreak in Rome, Italy: Broad Spectrum IncC Plasmids Spread the NDM Carbapenemase within the Hospital
Source: Antibiotics (Basel). 2023 May 22;12(5):943. doi: 10.3390/antibiotics12050943 (PMC10215782; doi:10.3390/antibiotics12050943)
Supplement: Supplementary file 1 [file antibiotics-12-00943-s001.zip › Table S1 and Figure S1.pdf]

Table S1: Clinical information

Patient	Age/ sex	COVID-19	Lenght of stay (days)	CM	Previous (90-d) hospitalization	Previous (90-d) antibiotic therapy	Previous (90-d) carbapenem therapy	Ward of isolation	Presence of NDM microorganism in the ward	Type of sample (first isolation)	Previous NDM colonization	Infection/ colonization	Antibiotic therapy for NDM infection	Outcome
**Pt#1**	52/M	Yes	33	None	Yes	Yes	No	COVID-19 ICU	Yes	Rectal swab	No	Colonization	NA	Death
**Pt#2**	50/M	Yes	23	None	No	Yes	No	COVID-19 ICU	Yes	Rectal swab	No	Colonization	NA	Death
**Pt#3**	65/M	No	37	None	Yes	Yes	No	General ICU	No	TBA	No	Infection (VAP, UTI)	CZA+AZT	Survived
**Pt#4**	36/M	Yes	168	HTN	No	Yes	No	COVID-19 ICU	Yes	Rectal swab	Yes	Infection (VAP, UTI)	CZA+AZT	Survived

CM: comorbidity; HTN: hypertension; ICU: Intensive Care Unit; TBA: tracheal aspirate; VAP: ventilator associated pneumonia; UTI: Urinary Tract Infection; CZA: ceftazidime/avibactam; AZT: aztreonam.

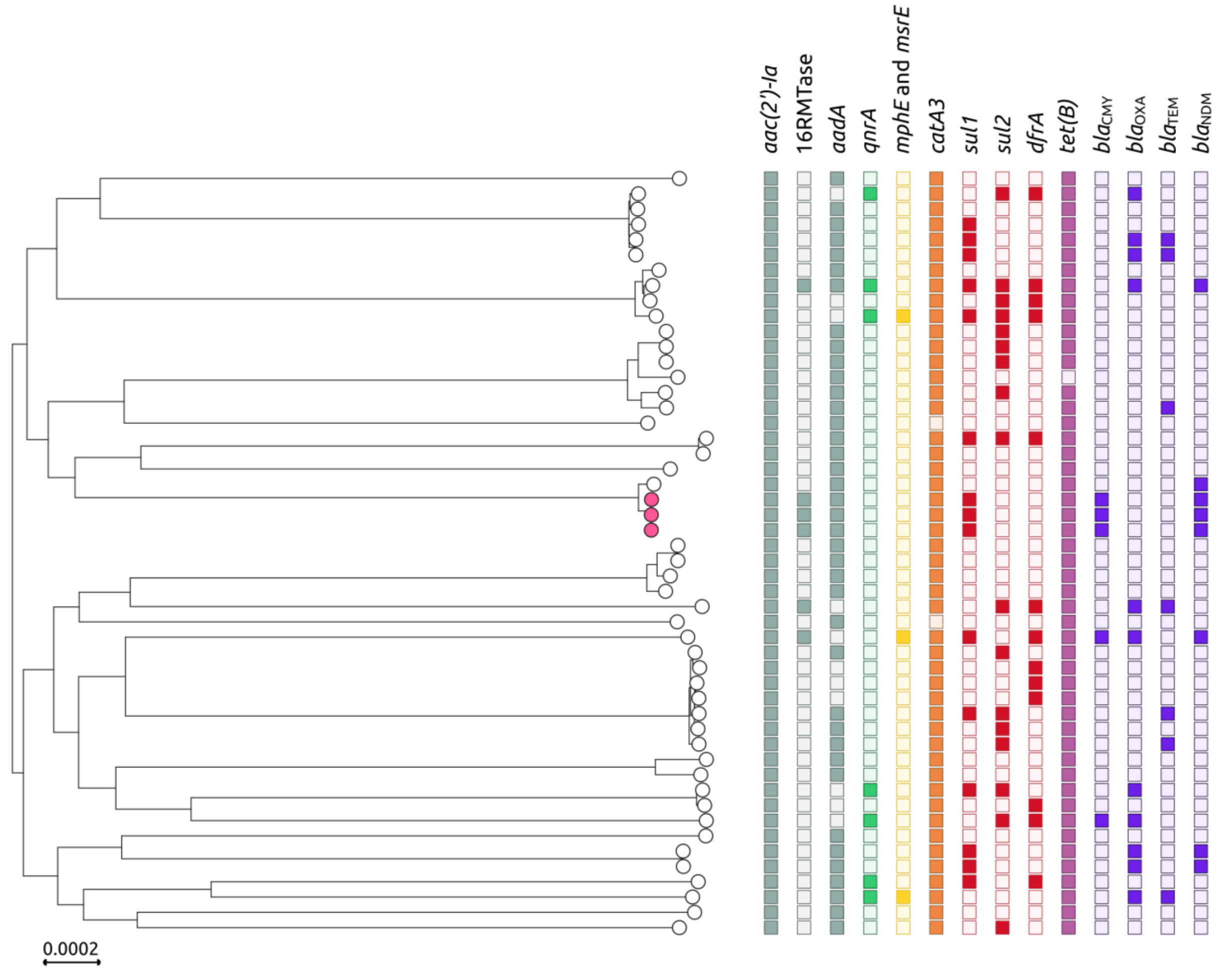

Figure S1. Phylogenetic tree of *Providencia stuartii*. Phylogenetic tree of 50 *Providencia* genomes (57 retrieved from the NCBI RefSeq database and 3 from this study) based on the concatenation of 2,065 core genes. Metadata represent the various resistance genes carried by the strains, and are color-coded according to the antibiotic class. The three isolates sequenced in this study are indicated by magenta dots.
